# Biochemical and Epigenetic Modulations under Drought: Remembering the Stress Tolerance Mechanism in Rice

**DOI:** 10.3390/life13051156

**Published:** 2023-05-10

**Authors:** Suresh Kumar, Karishma Seem, Trilochan Mohapatra

**Affiliations:** 1 Division of Biochemistry, ICAR-Indian Agricultural Research Institute, New Delhi 110012, India; 2 Indian Council of Agricultural Research, New Delhi 110001, India

**Keywords:** rice, terminal drought, 5-methylcytosine, stress memory, transgenerational memory

## Abstract

A plant, being a sessile organism, needs to modulate biochemical, physiological, and molecular responses to the environment in a quick and efficient manner to be protected. Drought stress is a frequently occurring abiotic stress that severely affects plant growth, development, and productivity. Short- and long-term memories are well-known phenomena in animals; however, the existence of such remembrance in plants is still being discovered. In this investigation, different rice genotypes were imposed with drought stress just before flowering and the plants were re-watered for recovery from the stress. Seeds collected from the stress-treated (stress-primed) plants were used to raise plants for the subsequent two generations under a similar experimental setup. Modulations in physio-biochemical (chlorophyll, total phenolics and proline contents, antioxidant potential, lipid peroxidation) and epigenetic [5-methylcytosine (5-mC)] parameters were analyzed in the leaves of the plants grown under stress as well as after recovery. There was an increase in proline (>25%) and total phenolic (>19%) contents, antioxidant activity (>7%), and genome-wide 5-mC level (>56%), while a decrease (>9%) in chlorophyll content was recorded to be significant under the stress. Interestingly, a part of the increased proline content, total phenolics content, antioxidant activity, and 5-mC level was retained even after the withdrawal of the stress. Moreover, the increased levels of biochemical and epigenetic parameters were observed to be transmitted/inherited to the subsequent generations. These might help in developing stress-tolerant crops and improving crop productivity under the changing global climate for sustainable food production and global food security.

## 1. Introduction

Rice is one of the stable food crops that provide food to about half of the global population [1]. Being a fat-free, gluten-free, cholesterol-free food, and low in sodium, rice is a highly beneficial component of a healthy diet for a growing population suffering from coronary artery disease, heart disease, blood pressure, and celiac disease [2]. Rice production would require an increase of 60% to feed the burgeoning global population by 2050 [3]. Growing populations, industrialization, urbanization, and climatic changes have aggravated environmental changes and their effects on crop production. Poor/erratic rainfall and lower precipitation (drought conditions) negatively affect rice productivity, which raises serious concerns regarding global food security. Rice cultivation by transplantation consumes plenty of fresh water, resulting in a limited availability of water for the irrigation of other field crops, particularly in the year of low rainfall [4]. Though the severity of abiotic stress depends upon several factors [5], more than one-third of the cultivated lands are affected by drought stress of varying intensities, which adversely affects biochemical, physiological processes, and the productivity of the plant [6]. Due to the decreasing availability of fresh water and decreasing yielding potential of crops, it has become important to enhance crop productivity under changing climatic conditions along with enhancing water productivity [7]. Although plants have evolved different mechanisms to cope with environmental stresses, the severity of stress aggravates yield losses.

Drought tolerance is a complex/multigenic trait requiring complementary effects of biochemical, physiological, and molecular mechanisms in protecting the plant. Among the various regulatory processes crucial for stress tolerance/adaptation in plants, the epigenetic modification-mediated regulation of gene expression has been reported to play important roles in managing environmental stress tolerance as well as evolutionary development [8,9]. It has also been suggested that plants may remember the strategies they adopted to cope with the stress deploying antioxidant defense, accumulation of osmolytes, expression of stress-associated genes, and epigenetic modifications, which enable quick and efficient responses to the reoccurrence of stress [10,11,12,13]. Oxidative burst during drought stress is mitigated by the integrated actions of enzymatic and non-enzymatic antioxidants, free radical scavenging, osmolytes, secondary metabolites, etc. [14]. Plants trigger biochemical, physiological, genetic, and epigenetic mechanisms to mitigate the adverse effects of drought stress [15,16]. Drought stress, particularly at the reproductive (panicle initiation) stage, severely reduces the yield of crops. Upon repetitive exposure to drought stress, some of the regulatory factors maintain their levels sufficiently high to keep themselves ready for defensive action [17]. Such a proactive defensive measure, named stress memory, helps to improve the responses of plants to the reoccurrence of stress [18,19].

Though the basic mechanism of stress memory in plants has not yet been fully understood, some of the reports on biochemical/metabolic and/or epigenetic modifications indicate their important roles in short- as well as long-term memories in plants [15,17,19]. Biochemicals such as proline, phenolic compounds, antioxidants, and secondary metabolites help to mitigate the adverse effects of environmental stresses [15,20,21,22,23,24]. Antioxidant potential had long been correlated with tolerance to various abiotic stresses [25,26,27,28,29]. The equilibrium between the generation of reactive oxygen species (ROS) and their scavenging is mediated by the enzymatic and non-enzymatic antioxidant defense systems. Secondary metabolites/phenolic compounds are synthesized/accumulated in response to abiotic stresses which take part in defense responses [22]. Phenolic compounds help to minimize the oxidation of vital biomolecules in the cell by reacting with free radicals, chelating metal ions, and acting as ROS scavengers [21]. The biosynthesis/accumulation of proline in plant tissue under abiotic stress helps to mitigate the adverse effects of stress. Free proline serves as a signaling molecule, affecting mitochondrial function, cell proliferation, and the expression of stress-responsive genes [24]. Proline also acts as an osmolyte, metal ion chelator, and antioxidative defense molecule that helps to maintain osmotic balance, membrane integrity, and minimize oxidative (ROS) burst under stress [14,15]. The estimation of proline content in plant tissue has been used as an established biochemical parameter for assessing the abiotic stress tolerance ability of plants [30,31] and it is speculated to play an important role in short- as well as long-term stress memory [32].

Epigenetic modifications (DNA methylation, histone modifications, and/or changes in small-RNA biogenesis) are reported to play important roles in affecting gene expression, particularly under environmental stresses [33,34,35,36,37]. Mitotic transmission, as well as meiotic transmission of epigenetic modifications in plants, has also been speculated to play a role [38,39]. It has recently been demonstrated that plants remember the strategies used to cope with the stress, and they deploy them to respond quickly and more efficiently in order to protect themselves from the reoccurrence of the stress [12,18,40].

The transgenerational inheritance of epigenetic marks requires their passage through the germline cells without getting erased by the cellular/surveillance mechanisms in animals [41]. However, plants do not possess germline cells (as in animals) but produce gametes from meristematic cells relatively later in their life cycle. Thereby, some of the epigenetic changes acquired during the vegetative stage might be inherited through mitotic as well as meiotic cell divisions [18,40]. Evidence also indicates that epigenetic modifications are part of the stress memory/adaptation mechanism in plants [42]. Without having a nervous system, plants are reported to memorize some of the events probably through transcription factors (TFs), post-translational modifications, phytohormones, metabolites, epigenetic modifications, chromatin architecture, etc. [12,43]. An alteration in the transcription activation mark (H3K4me3) was reported to play a role in transcriptional stress memory under drought [44,45]. Small-RNA was also reported to play a role in stress memory on repeated drought stress in Arabidopsis [40]. Arabidopsis mutants (lacking de novo DNA methylation, production of siRNAs) indicated the involvement of transcriptional and post-transcriptional regulation of gene expression under low humidity stress [46]. About 70% of the stress-induced epigenetic alterations revert to the original state on reversal of the stressful conditions, but part of them might be inherited as epigenetic stress memory [11,47]. A part of drought-induced DNA methylation was reported to be maintained in the subsequent generations under drought stress in rice [48]. However, several questions regarding the role/mechanism of epigenetic marks in keeping stress memory, their persistence, stability, and inheritance remain unanswered.

Studies are focusing on deciphering the mechanisms of stress priming/memory in plants [28,49,50]. Enhanced root growth/biomass was reported in *Polygonum persicaria* progenies of the parent exposed to drought stress [51]. Arabidopsis plants exposed to repetitive dehydration stress showed slower wilting compared to that of the unprimed plants [40]. Similarly, an improved retention of water was reported due to repetitive drought stress in *Zea mays* [52]. While enhanced drought tolerance was reported before anthesis due to stress priming in wheat [53], improved drought tolerance in potatoes was also reported [49]. Moreover, drought priming was reported to improve the productivity of olive under drought stress [54]. A similar effect of the priming was reported in bread wheat [55].

Therefore, to have better insights into the existence of stress memory mediated by biochemical and/or epigenetic interventions in modulating plant responses to drought stress, an experiment on drought stress tolerance in rice was executed using different rice genotypes [Sahbhagidhan 1, Nagina 22, IR64-DTY_1.1_ (drought-tolerant) and IR64 (drought-sensitive)]. Rice plants were imposed with drought stress just before the initiation of flowering and the plants were re-watered after the collection of tissue samples. The experiment was repeated for three years. In previous studies, the differential expression of genes in the leaf of N22 was reported to play a more important role in the genetic plasticity of rice in response to varying environmental conditions [4,16]. Hence, in this study, we focused on leaf tissues only to observe certain physio-biochemical (chlorophyll content, lipid peroxidation, antioxidant activity, phenolics content, proline content) and epigenetic (DNA methylation) parameters. Protective physio-biochemical and epigenetic changes in response to drought stress, partial reversal on recovery from stress, and the inheritance of the retained component to the subsequent generations indicate the existence of biochemical as well as epigenetic stress memory, which is helpful regarding tolerance to drought stress in rice. Thus, the present study provides evidence for the transgenerational inheritance of adaptive changes and their involvement in stress tolerance/acclimatization in plants.

## 2. Materials and Methods

### 2.1. Plant Materials and Drought Stress Imposition

Mature and healthy seeds of contrasting rice cultivars/genotypes [Sahbhagidhan 1 (SBG), Nagina 22 (N22), IR64-DTY_1.1_ (DTY1.1) drought-tolerant, and IR64 (drought-sensitive)] were used to grow nurseries. Three (twenty-five-day-old) seedlings were transplanted in 12″ plastic pots filled with soil in nine replications. The plants were grown in a net-house under natural conditions during July–October (*Kharif* season). Plants/pots maintained as control were watered on an alternate days, and they were not imposed with water-deficit stress, while drought stress was imposed on them by withholding watering/irrigation for 4–5 days just before the initiation of flowering (terminal drought stress, 65 days after transplantation of N22, in the middle of September). When the leaves of the rice plants started wilting, the soil moisture content (SMC) reduced to 1/4, and the relative water content (RWC) of the leaf reduced to ~58%; then, leaf tissues were collected from randomly selected pots in six replications (each replication comprised the tissues from all three plants in a pot) for physio-biochemical and epigenetic analyses. After the collection of leaf tissues from the drought-treated rice plants, the plants were irrigated immediately and allowed to grow under normal/irrigated conditions. Once the plants recovered from the stress for 10 days, leaf tissues were collected again as the after-recovery sample. In our earlier findings [4,16], we observed a major role of the leaf in the acclimatization of rice plants to the changing environmental conditions; hence, we restricted our analyses to the leaf tissues of the rice genotypes.

### 2.2. Estimation of Soil Moisture and Relative Water Content

To estimate the level of drought stress and water content in the soil, soil moisture content (SMC) was determined by the gravimetric method in three biological replications as described earlier [4], using the soil samples collected at a depth of 5 cm from the pots. The soil sample was placed in a pre-weighed Petri plate and the weight of the soil was recorded immediately. The soil samples were dried at 60 °C in an oven until the constant dry weight (DW) of the soil sample was achieved. The SMC was calculated using the following formula:

SMC (%) = [(weight of wet soil − weight of dried soil) ÷ (weight of dried soil)] × 100

The relative water content (RWC) of the leaf was estimated by collecting 10 cm leaf tissues (in three biological replications) following the method described earlier [4]. The tissues were cut into small (5 mm) pieces in a pre-weighed Petri plate covered with a lid to record the fresh weight (FW) of the leaf. The Petri plate was filled with distilled water and stored at room temperature for 4 h to obtain a turgid condition of the leaf. The turgid weight (TW) of the leaf was recorded, and tissues were blot-dried and then air-dried in an oven at 60 °C until a constant weight (DW) was achieved. The RWC of the leaf was calculated using the following formula:

RWC (%) = [(FW − DW) ÷ (TW − DW)] × 100

### 2.3. Estimation of Chlorophyll Content

The total chlorophyll content in the leaf (0.5 g) of different rice genotypes grown under control and drought stress conditions was estimated in three biological replications using the dimethyl sulfoxide (DMSO) method, as described elsewhere [15]. Chlorophyll was extracted from the fresh leaf tissues by dipping them in 20 mL DMSO in a tube, incubating them in the dark for 4 h at 4 °C, and repeating the process once again. The extracts were pooled together and the final volume was made up to 50 mL with DMSO. The absorbance of chlorophyll extract was recorded spectrophotometrically at 645 and 663 nm using DMSO as a blank in three technical replications. The total chlorophyll content in leaf tissue was calculated on a dry weight (DW) basis using the following formula:

Total chlorophyll content (mg/g DW) = DMI [{(20.2 × A_645_) + (8.02 × A_663_)} × (V ÷ W)]

where DMI = dry matter index (fresh weight ÷ dry weight) of leaf tissues, V = volume (mL) of DMSO used to extract the tissue sample, W = weight (mg) of the sample tissue.

### 2.4. Estimation of Lipid Peroxidation

The lipid peroxidation in leaf tissues was determined, in terms of malondialdehyde (MDA) level, using the thiobarbituric acid reaction described earlier [15,28]. The tissue extract was prepared by grinding 1.0 g fresh leaf tissue (in three biological replications) in 20 mL TCA (0.1%) solution followed by centrifugation for 10 min at 12,000 rpm. Then, 1 mL of the supernatant was reacted with 4 mL TCA solution containing 0.6% thiobarbituric acid. The reaction mixture was heated at 95 °C for 30 min, cooled on ice, and then centrifuged for 10 min at 12,000 rpm. The absorbance of the reaction mix was recorded at 532 and 600 nm in three technical replications, and the MDA level in leaf tissues was calculated with the help of an extinction coefficient of 155 mM^−1^ cm^−1^ using the following formula:

MDA level (nmol) = ΔA_(532–600 nm)_ ÷ (1.56 × 10^5^)

### 2.5. Estimation of Antioxidant Activity

Antioxidant activity in the tissue extracts was measured using the stable DPPH (2,2-diphenyl-1-picrylhydrazyl) radical scavenging method as described earlier by Kaur et al. [24]. Fresh leaf tissues (1.0 g) were ground into a fine powder (in three biological replications) and extracted with 10 mL ethanol (90%) by constant shaking for 48 h at room temperature. The extract was centrifuged at 13,000 rpm for 10 min and the supernatant was collected for the estimation of antioxidant activity. An alcoholic solution (0.5 mL) of DPPH radical (0.2 mM) was added to 100 μL of sample extract, mixed vigorously, and incubated in the dark for 45 min. Finally, absorbance (A_517_) was recorded (in three technical replications) and the scavenging of DPPH was calculated using the following formula:

DPPH Scavenging (%) = [(A_0_ − A_1_) ÷ A_0_] × 100

where A_0_ is the absorbance of the control reaction and A_1_ is the absorbance of the sample at 517 nm. The inhibitory concentration at 50% (IC_50_, extract concentration that causes 50% scavenging of DPHH radical) was also determined experimentally.

### 2.6. Estimation of Total Phenolics Content

To determine the total phenolics content (TPC) in leaf tissues collected from different rice genotypes, the procedure of Singleton et al. [56] was used. About 1.0 g of fresh leaf tissue was ground into a fine powder (in three biological replications) and homogenized in 20 mL of trichloroacetic acid (0.1%). The homogenate was centrifuged at 12,000 rpm for 10 min at room temperature; 0.5 mL of the aqueous extract was mixed with 20.5 mL of Folin–Ciocalteu reagent (10% *v*/*v*) and 2 mL of 7.5% sodium carbonate. The reaction mixture was incubated for 40 min at 45 °C and absorbance (A_765_) was recorded in 3 technical replications. A phenol standard curve for TPC was prepared (using 0–80 μg gallic acid) in terms of the gallic acid equivalent of the sample tissue.

### 2.7. Estimation of Proline Content

The proline content in the leaf was estimated in three biological and three technical replications following the method described earlier [15,31], using Ninhydrin reagent. The proline–ninhydrin chromophore was extracted with 4.0 mL toluene and the absorbance was recorded spectrophotometrically at 520 nm. The proline content in the sample tissue was determined from a standard curve (0–25 µg), calculated using the following formula, and expressed on a dry weight basis.

Proline (μmol/g DW) = DMI [(μg Proline/mL × mL Toluene) ÷ (115.5 μg/μM)] ÷ [(g sample) ÷ 5]

where DMI = Dry matter index (fresh weight ÷ dry weight) of leaf tissues.

### 2.8. Estimation of Genome-Wide 5-Methylcytosine Content

Genome-wide DNA methylation (5-mC) content in the leaf of a pair of contrasting rice (N22 and IR64) genotypes was estimated using MethylFlash Methylated DNA Quantification (Colorimetric) kit (Epigentek). Genomic DNA (100 mg), isolated from leaf tissues in three replications using a Genomic DNA isolation kit (Qiagen), was used for the detection of 5-mC content deploying a specific monoclonal antibody (along with a detection/secondary antibody). Negative and positive DNA controls provided in the kit were used for preparing the standard curve to quantify the 5-mC content. The colorimetric estimation of 5-mC content was performed using a microplate reader following the manufacturer’s instructions. The sample genomic DNA was first coated on the surface of the wells (in triplicate) of the assay plate. After color development, following the manufacturer’s instructions, absorbance was measured at 450 nm using a microplate reader. The quantification of 5-mC (%) was performed by plotting the standard curve and using the following formula:

$$5-mC\;\left(\%\right)=\frac{\left(\mathrm{Sample}\;\mathrm{OD}\;-\mathrm{ME3}\;\mathrm{OD}\right)÷S}{\left[\left(\mathrm{ME4}\;\mathrm{OD}-\mathrm{ME3}\;\mathrm{OD}\right)\times 2\right]÷p}\times 100$$

where S is the amount of input sample genomic DNA in ng; *p* is the amount of input positive control (ME4) in ng; ME3 is the negative control; ME4 is the positive control; and 2 is used to normalize 5-mC in the positive control as the positive control contains only half of 5-mC.

### 2.9. Statistical Analysis

The experiments performed for these investigations were carried out with three (or more) replications. Statistical analysis was performed using analysis of variance (ANOVA), post hoc Tukey test and/or Duncan’s multiple range test (DMRT) at *p* ≤ 0.05 to compare the means of treatments for their significance. The standard deviation (±SD) was calculated and represented as an error bar.

## 3. Results

### 3.1. Effect of Drought Stress/Lowered Soil Moisture Content on Relative Water Content of Leaf

**Figure 1:**
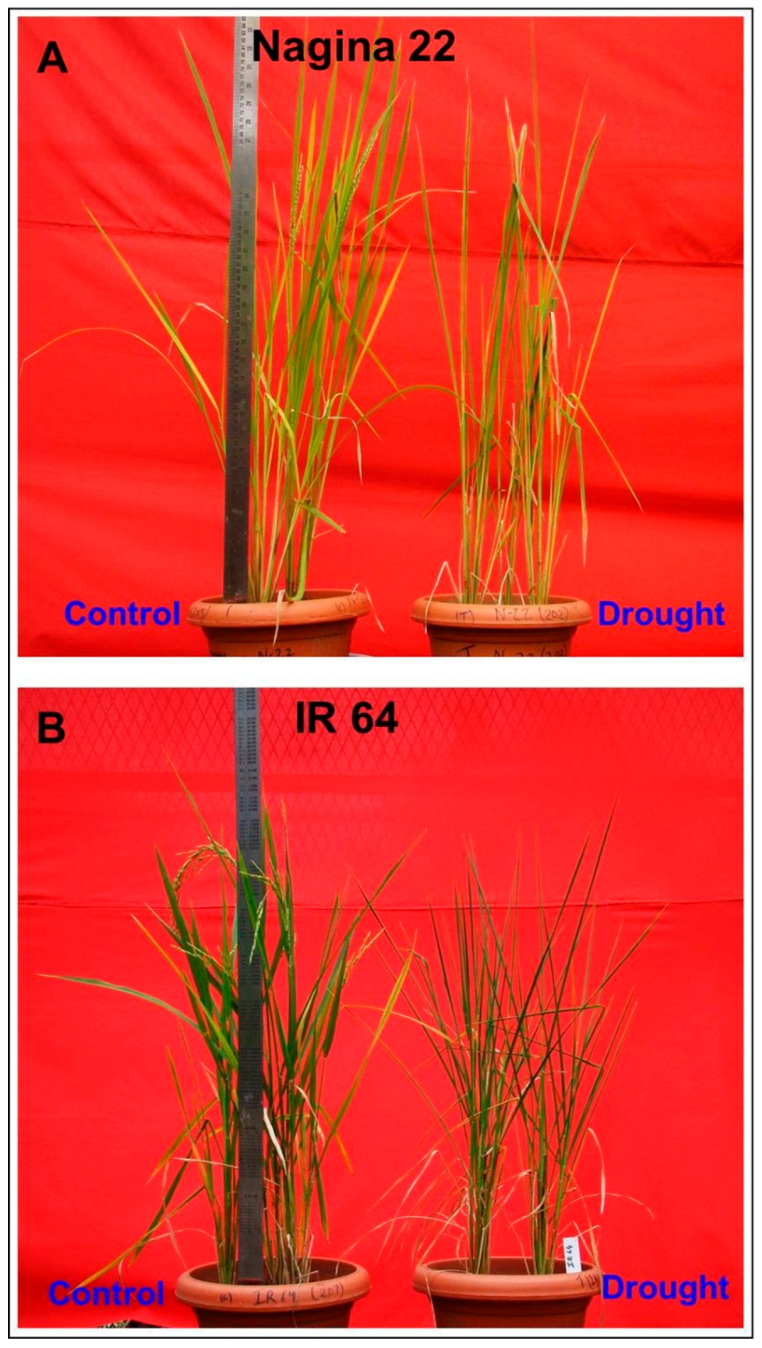
Representative picture of a pair of contrasting rice genotypes grown under control and drought stress, imposed with terminal drought stress. (**A**) Nagina 22 (N22), (**B**) IR 64 rice genotype.

The imposition of drought stress, by withholding irrigation until the soil moisture content dropped down to ~6% (compared to ~24% in the control/well-watered pots), resulted in a greater reduction in RWC (58 ± 1%) in the leaf of IR64 compared to that (61 ± 1%) in the leaf of N22. A noticeable/morphological symptom of drought stress could be visualized in the form of the rolling/wilting of leaves (Figure 1).

### 3.2. Effect of Terminal Drought Stress on Chlorophyll Content

**Figure 2:**
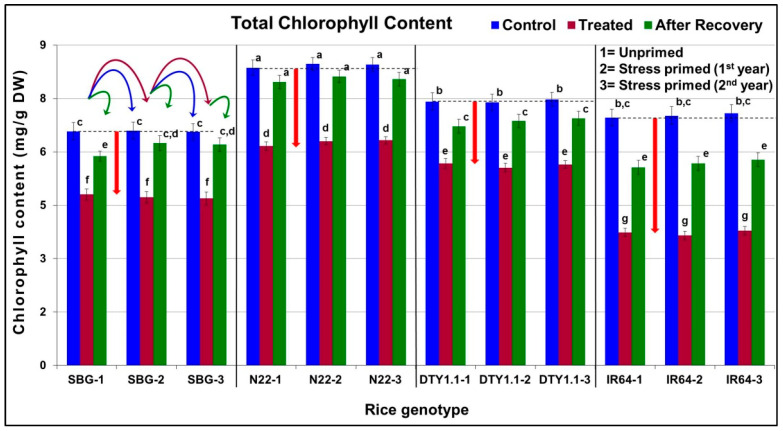
Diagrammatic representation of the effect of drought stress on chlorophyll content in leaves of different rice genotypes. The downward arrow (↓) shows the extent of reduction in chlorophyll content on stress imposition in the rice genotypes on first exposure to the stress. SBG = Sahbhagidhan 1, N22 = Nagina-22, DTY1.1 = a Near Isogenic Line of IR64 harboring *qDTY_1.1_*, the drought-tolerant genotypes; IR64, drought-sensitive genotype. Data present mean value (*n* = 3). Means followed by different lower-case letters are significantly different (*p* < 0.05). The error bar represents the standard deviation (±SD). The different color curved arrows in the first panel indicate the source of seeds/plants used for growing plants/treatments over three generations for all four genotypes.

The stress caused a 9–16% reduction in the total chlorophyll content in the leaves of drought-tolerant (SBG, N22, and DTY1.1) genotypes, while the reduction was >25% in the leaves of IR64 (Figure 2). More importantly, a significantly higher (92–98%) recovery in total chlorophyll content was observed in the leaves of drought-tolerant genotypes 10 days after re-watering the plants compared to that in the drought-sensitive genotype. Although a similar trend of reduction in total chlorophyll content on drought stress was observed in all the drought-tolerant genotypes, the recovery in chlorophyll content was better in the stress-primed plants in the second and third years/generations. However, the recovery in chlorophyll content was significantly lower (~85%) in the stress-primed plants of IR64, even in the second and third years/generations (Figure 2).

### 3.3. Effect of Drought Stress on Lipid Peroxidation in Leaf

**Figure 3:**
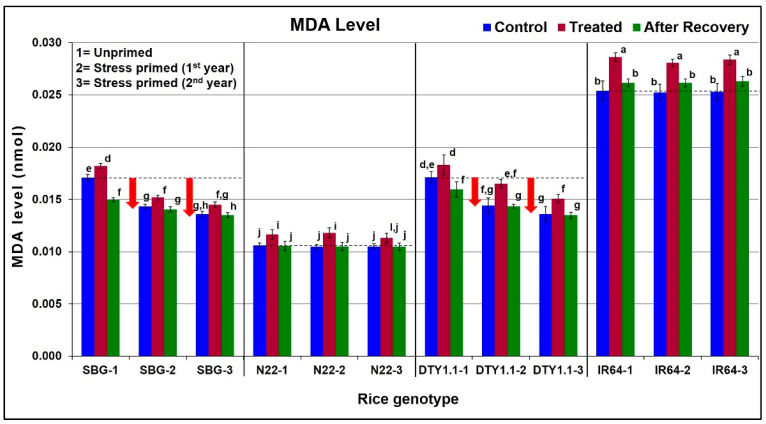
Diagrammatic representation of the effect of drought stress on malondialdehyde (MDA) level (lipid peroxidation) in leaves of different rice genotypes. The downward arrow (↓) shows reduced MDA levels under control conditions in stress-primed plants of the drought-tolerant genotypes. Data present the mean value (*n* = 3). Means followed by different lower-case letters are significantly different (*p* < 0.05). The error bar represents the standard deviation (±SD).

The effect of drought stress on lipid peroxidation in leaf tissues was assessed based on the changes in MDA level under the stress, which significantly increased (6–12%) in the rice genotypes under the stress. However, the increase was significantly higher (12%) in the drought-sensitive (IR64) genotype compared to that (6–10%) in the leaves of the drought-tolerant (SBG, N22, and DTY1.1) genotypes (Figure 3). Interestingly, the MDA level was maintained considerably lower even under control conditions in the leaves of the drought-tolerant (SBG, N22, and DTY1.1) genotypes compared to that in the leaves of IR64. Although a significant (~10%) increase in MDA level was recorded on drought stress imposition, the level (lipid peroxidation) reduced to a lower extent after recovery from the stress in the drought-tolerant genotypes. However, a much higher (~48%) level of MDA was recorded in the leaf of IR64 even under control conditions, which increased significantly (~67%) upon drought stress imposition. However, even after recovery, the MDA level could not return to the normal level. More interestingly, the MDA level (0.0106 nmol) in the leaves of N22 was the lowest (even under drought stress), while the basic MDA level (under control conditions) in the leaves of stress-primed plants of SBG and DTY1.1 in the first and second year showed a diminishing pattern (Figure 3).

### 3.4. Effect of Drought Stress on Antioxidant Potential in Leaves of Different Rice Genotypes

**Figure 4:**
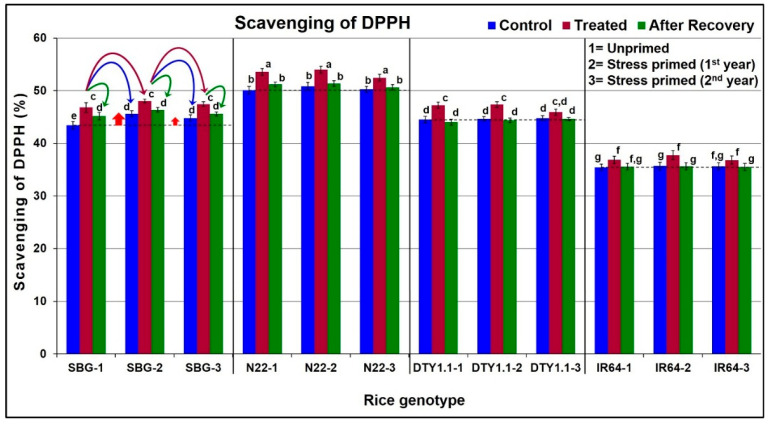
Diagrammatic representation of the effect of drought stress on scavenging of DPPH in leaves of different rice genotypes. The upward arrow (↑) shows increased DPPH level under control conditions in the stress-primed plants of the drought-tolerant genotype. Data present the mean value (*n* = 3). Means followed by different lower-case letters are significantly different (*p* < 0.05). The error bar represents the standard deviation (±SD). The different color curved arrows in the first panel indicate the source of seeds/plants used for growing plants/treatments over three generations for all four genotypes.

A significant (2–6%) increase in the scavenging of DPPH was observed in all four rice genotypes under drought stress. However, the basic level of DPPH was considerably higher, and a maximum (6%) increase was observed in the leaves of N22 compared to that in the leaves of IR64 (drought-sensitive genotype) (Figure 4). Interestingly, the DPPH scavenging level was maintained higher even after recovery from the stress in the stress-primed plants (in the first and second year) of the SBG (drought-tolerant) genotype. Moreover, the DPPH scavenging level in the leaves of SBG was higher even under control conditions in the subsequent generations of stress-primed plants (of the first and second year) (Figure 4).

**Figure 5:**
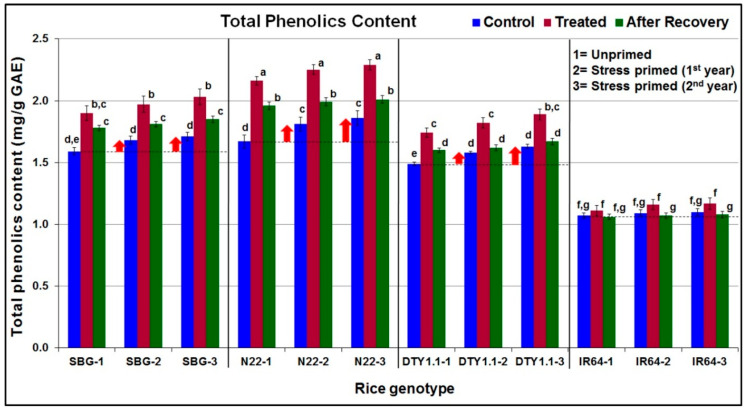
Diagrammatic representation of the effect of drought stress on total phenolics content in leaves of different rice genotypes. The upward arrow (↑) shows increased total phenolics content in leaves under control conditions in the stress-primed plants of the drought-tolerant genotypes. SBG = Sahbhagidhan 1, N22 = Nagina-22, DTY1.1 = a Near Isogenic Line of IR64 harboring *qDTY_1.1_*, drought-tolerant genotypes; IR64, drought-sensitive genotype. Data present the mean value (*n* = 3). Means followed by different lower-case letters are significantly different (*p* < 0.05). The error bar represents the standard deviation (±SD).

A significant (4–26%) increase in total phenolics content in the leaves of all four rice genotypes was observed; however, the maximum increase was recorded in N22 under stress. Increased TPC was also observed in the other two (SBG and DTY1.1) drought-tolerant genotypes, but no significant increase was recorded in the case of IR64 under stress (Figure 5). Stress priming caused increased TPC in the plants of the second year even under control conditions. TPC further increased significantly (15–24%) following the drought stress imposition on the tolerant genotypes. The increase in TPC content in the leaves of IR64 upon drought stress imposition was not high, and no effect of stress priming was observed in IR64 either (Figure 5).

**Figure 6:**
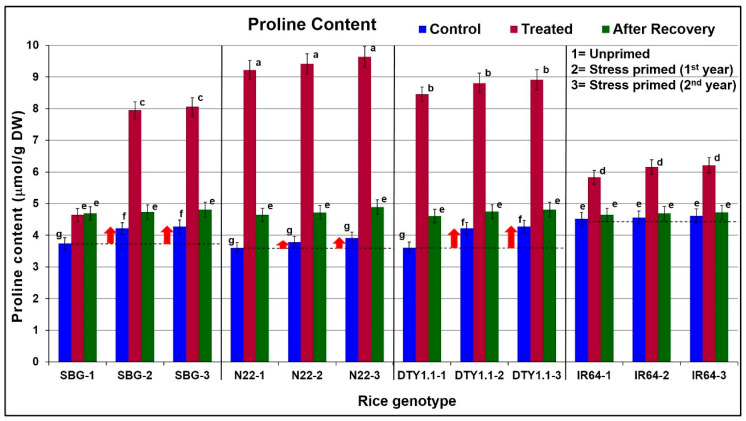
Diagrammatic representation of the effect of drought stress on proline content in leaves of different rice genotypes. The upward arrow (↑) shows increased total proline content in leaves under control conditions in the stress-primed plants of the drought-tolerant genotypes. SBG = Sahbhagidhan 1, N22 = Nagina-22, DTY1.1 = a Near Isogenic Line of IR64 harboring *qDTY_1.1_*, drought-tolerant genotypes; IR64, drought-sensitive genotype. Data present the mean value (*n* = 3). Means followed by different lower-case letters are significantly different (*p* < 0.05). The error bar represents the standard deviation (±SD).

Though a significant (>24%) increase in proline content in leaves was observed in the rice genotypes following drought stress imposition, it increased up to 2.5-fold in the leaves of the drought-tolerant genotypes (SBG, N22, and DTY1.1). Although the basal proline content (under unstressed conditions) of N22 was comparatively lower, the increase in proline content on drought stress imposition was the highest (Figure 6). With a minimal increase in proline content on drought stress imposition in the first year, the effect of stress priming in IR64 was not significant (Figure 6).

### 3.5. Change in 5-Methylcytosine (5-mC) Content on Drought Stress in Rice

**Figure 7:**
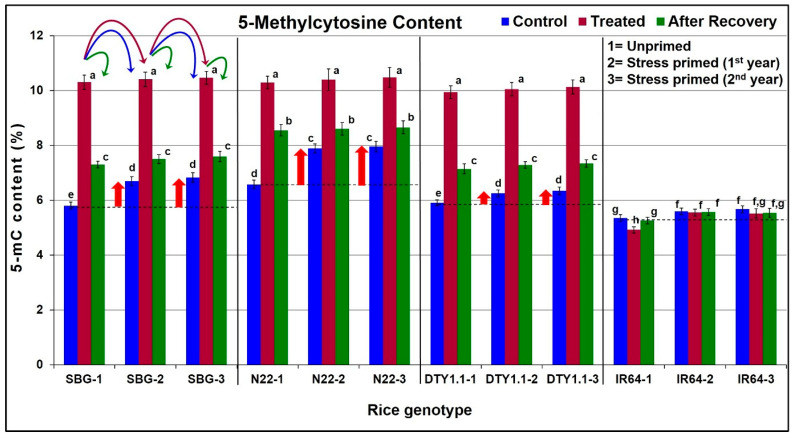
Diagrammatic representation of the effect of the reproductive stage drought stress on 5-methylcytosine (5-mC) content in rice genotypes and its heritability across the generation. The upward arrow (↑) shows increased 5-methylcytosine content in leaves under control conditions in the stress-primed plants of the drought-tolerant genotypes. SBG = Sahbhagidhan 1, N22 = Nagina22, DTY1.1 = Near-isogenic line of IR64 harboring *qDTY_1.1_*, drought-tolerant genotypes; IR64, drought-sensitive genotype. Data present the mean value (*n* = 3). Means followed by different lower-case letters are significantly different (*p* < 0.05). The error bar represents the standard deviation (±SD). The different color curved arrows in the first panel indicate the source of seeds/plants used for growing plants/treatments over three generations for all four genotypes.

In the present investigation, the changes in genome-wide 5-mC content in leaves of a pair of contrasting rice (N22 and IR64) genotypes, subjected to reproductive stage drought stress followed by recovery from the stress across the generations, were investigated. We observed a significant (~4.5%) increase in the overall 5-mC content in the drought-tolerant genotypes (Figure 7). Interestingly, a significant (~0.5%) decrease in the global 5-mC content was observed in the leaves of IR64 (drought-sensitive genotype) on the imposition of drought stress. Moreover, even after recovery from the stress, 5-mC content was observed to be higher compared to the level observed under the control condition. This indicates the retention of a part of the increased 5-mC content (~1.2%) after the withdrawal of the stress. More interestingly, in the next generation of stress-primed plants, the 5-mC content under the control condition was significantly (~1.0%) higher than that observed under the control condition in the previous generation (but lower than that observed after the recovery). A similar trend of increased 5-mC content (compared to the 5-mC content under control in the first generation) was observed in the subsequent (third) generation under the control condition, which further increased upon stress imposition (Figure 7). Such a trend was observed in all three (SBG, N22, and DTY1.1) stress-tolerant genotypes but not in the stress-sensitive (IR64) genotype. Though a significant (~0.5%) decrease in 5-mC content was observed upon stress imposition in IR64, the trend was not carried forward in the subsequent generations.

A part (~25%) of the increased methylation level was retained even after the withdrawal of the stress in the drought-tolerant genotypes, with a maximum in the case of N22, particularly in the first year of stress imposition (Figure 7). However, a clear difference in the modulation of 5-mC content between N22 and its near-isogenic line (DTY1.1) was observed. Although the increase in 5-mC content on drought stress imposition was the same, the amount of increased 5-mC retained after recovery, as well as the increased 5-mC content in the subsequent generation under control, was comparatively lower in DTY1.1.

## 4. Discussion

Due to global climate change, the frequency and distribution of rainfall are becoming scarce and erratic day by day leading to the frequent occurrence of drought stress [57]. Though rice is a water-loving (semi-aquatic) crop, its water-use efficiency is low compared to many other cereal crops [7,24,57]. Therefore, the genetic improvement of rice for enhanced tolerance to drought stress occurring at different developmental stages has become the need of the day. An identification of the mechanisms involved in drought stress tolerance and their potential usage in developing stress-tolerant genotypes need to be ensured. Although much has been explored about the biochemical basis of abiotic stress tolerance in plants, only a little has been studied about the regulatory mechanisms involved in terminal (reproductive stage) drought tolerance in rice. Moreover, the biochemical and epigenetic bases of abiotic stress memory have been poorly documented. The stress priming of plants has been reported to be regulated at transcription, translation, protein-phosphorylation, and metabolite levels [12]. Sugars, as well as lipid metabolites, were reported to play important roles in the adaptation of plants to environmental modulations [58,59]. Under the changing climate/unpredictable weather conditions, plants must modulate their physio-biochemical processes for their survival and better performance. Some plants have an inherent capability to adapt the changing environmental conditions by modulating their metabolic processes. However, they require memorizing the past episode(s) of environmental stress and the mechanism(s) used to overcome the stressful conditions. Stress priming has been reported to keep the plants ready for quicker/more efficient responses to the reappearance of stress [12,13]. For a plant to remember an episode of stress (stress memory), different mechanisms including morphological, physiological, biochemical, transcriptional, proteomic, and epigenetic modulations might be required [11,12]. While a short-term stress memory (within the generation) helps to improve tolerance to the stress (or induce cross-tolerance to other stresses) occurring subsequently within the generation [60,61,62], transgenerational (long-term) stress memory has been poorly understood in plants.

A reduction in the chlorophyll content and regaining it to a greater extent after 10 days following the withdrawal of the stress indicate better adaptive mechanisms of drought-tolerant genotypes under the stress (Figure 2). Approximately a 25% reduction in chlorophyll content in the leaves of N22 and DTY1.1 (drought-tolerant) genotypes compared to a ~50% reduction in the leaves of IR64 (drought-sensitive) on imposition of terminal drought stress confirmed the potential effects of *qDTY_1.1_* in maintaining higher chlorophyll content (compared to that in IR64) under the stress (Figure 2). Moreover, the extent of recovery in chlorophyll content (10 days after withdrawal of the stress) not only indicated the better performance of drought-tolerant genotypes but also the effects/benefits of stress priming. Our findings of a significant reduction in chlorophyll content in crop plants under drought stress corroborate the earlier findings [63,64]. A significantly lesser effect of stress priming on the recovery of chlorophyll content, after the stress, in IR64 (Figure 2) confirms the drought sensitivity of this genotype. In addition, a significant increase in lipid peroxidation (MDA level) under drought stress in the leaves of IR64 confirmed its vulnerability to the adverse effects of the stress. The increased MDA level due to abiotic stress in plants corroborates several earlier reports [15,28,65]. Moreover, the recovery of MDA level after the withdrawal of the stress (lowered down than that observed under control condition) and interestingly its decreasing level under the stress in the subsequent generations, particularly in the case of SBG-1 and DTY1.1 (Figure 3), indicate the existence of stress memory. A comparative analysis of the MDA level in DTY1.1 with that observed in N22 (the QTL donor) and IR64 (the recipient) indicates the important role of *qDTY_1.1_* in providing drought stress tolerance. The considerably lower MDA level maintained by N22 under all three conditions, and a minor but significant increase in MDA level under the stress and complete recovery (rollback) of MDA level after the withdrawal of the stress substantiate its multiple-stress tolerant nature reported earlier [66,67,68].

A significant increase in antioxidant activity (scavenging of DPPH) in all four rice genotypes under stress, maintaining increased antioxidant activity even after the withdrawal of the stress, and a higher DPPH scavenging activity under control conditions in the subsequent generations, particularly in the case of SBG (drought-tolerant genotype), indicate the role of biochemical stress memory in protecting the plants from drought stress in the subsequent generation (Figure 4). However, a comparative analysis of the antioxidant activity in the leaves of N22, DTY1.1, and IR64 indicated the role of *qDTY_1.1_* in considerably increasing antioxidant activity in DTY1.1 under stress, but the involvement of stress memory was not evident. The role of antioxidant activity in protecting plants from abiotic stresses has been well reported in different crop plants [15,24,28,69,70]. Similarly, the basal level (under control conditions) of TPC in drought-tolerant (SBG, N22, and DTY1.1) genotypes were maintained considerably higher compared to that in the drought-sensitive genotype (IR64). Moreover, the increase in TPC upon stress imposition, the preservation of a part of the increased TPC after recovery, and TPC being present even under control conditions in the subsequent generations of the drought-tolerant genotypes (while only a marginal increase in TPC in the leaf of IR64 was seen) (Figure 5) indicate its role in maintaining drought stress memory. A comparative analysis of TPC in the leaves of N22, DTY1.1, and IR64 indicated the role of *qDTY_1.1_* not only in maintaining higher TPC but also in the biochemical stress memory. Our findings on increased TPC under drought stress in the rice genotypes corroborate those reported earlier in different crops [24,28,71]. A considerable increase in the proline content of the leaves of the drought-tolerant (SBG, N22, and DTY1.1) genotypes under stress, and the preservation of a part of the increased proline content even after the 10-day recovery period showed the preparedness of the plants to the reoccurrence of stress. Moreover, the higher basal proline content in the leaves of the stress-primed drought-tolerant genotypes even under control conditions not only confirms its role in protecting the plants from stress but also indicates its important role in biochemical stress memory. Among the tested rice genotypes, IR64 (drought-sensitive genotype) showed the minimum increase in proline content under stress. The comparative analysis of proline content in the leaves of *qDTY_1.1_* donor (N22), recipient (IR64), and the NIL (DTY1.1) indicates the important roles of the QTL in accumulating proline under drought stress (Figure 6). Proline, as an osmolyte, metal ion chelator, and antioxidant molecule, helps to protect the plant from oxidative bursts under different abiotic stresses [14,15]. An enhanced antioxidant capacity due to proline accumulation was reported to be strongly associated with spikelet fertility, higher grain yield, and abiotic stress tolerance in rice [72]. All of these support our findings on the increased proline content in the leaves of drought-tolerant genotypes being responsible for protecting them from stress, as well as our hypothesis that stress priming/biochemical stress memory helps to protect the plants from recurring stress. Our findings on the effect of stress priming on abiotic stress tolerance in crop plants are in agreement with those reported earlier [50,73].

Our observations of a significant (~4.5%) increase in the global 5-mC content in the leaves of drought-tolerant genotypes, and, conversely, a significant (~0.5%) decrease in the drought-sensitive genotype (IR64) indicate the important roles of DNA methylation in the regulation of gene expression. The preservation of some of the increased 5-mC content 10 days after the withdrawal of the stress, and the increased 5-mC level even under the control condition in the subsequent generation indicate the important role of epigenetic modification in stress memory (Figure 7). A comparative analysis of the 5-mC content under control conditions with the increase in 5-mC content under drought stress showed that part of the increased 5-mC content was retained after the withdrawal of the stress. The modulations in the 5-mC content in the subsequent generation of N22 (QTL donor), IR64 (the recipient), and the NIL (DTY1.1) indicate a major role of *qDTY_1.1_* in drought tolerance through the epigenetic modification of DNA. As the genetic difference between IR64 and DTY1.1 is the presence of *qDTY_1.1_* in DTY1.1 (introgressed in IR64 genetic background from N22), the observed differences between IR64 and DTY1.1 in the biochemical parameters as well as in 5-mC content are due to the QTL. The retention of a part of the increased 5-mC content 10 days after the withdrawal of the stress, and a higher 5-mC content under control conditions in the next generation of stress-primed plants (than that observed in the previous generation under control), but lower than that observed after the recovery, provide evidence for the transmission of some epigenetic changes associated with drought stress tolerance across the generation; thus, this shows the heritability of the acquired adaptive change in plants. Our findings on the involvement of DNA methylation in epigenetic stress memory are in agreement with those reported earlier by Zheng et al. [48] and reviewed by Liu et al. [50]. However, the period/intensity of initial stress, the recovery periods in the case of cyclic stresses, and the mechanism(s) involved in eliciting/resetting the stress memory are crucial for establishing/reinforcing the memory [50].

However, extensive efforts would be required to understand the dynamics of DNA methylation and its role in plant growth, development, and stress tolerance [9]. Moreover, for improving abiotic stress tolerance in crops, we generally pay more attention to the stress-induced differentially methylated regions (DMRs) compared to those on the alterations in global methylation levels. The identification/mapping of epigenetic quantitative trait loci (epi-QTLs) might be more helpful in pointing out the causal DMRs involved in stress tolerance and stress memory in plants. Several DMRs have been identified as epi-QTLs controlling growth, development, and plasticity under abiotic stress in *ddm1* epi-RILs [74,75]. As a preliminary report, the present study demonstrates the inheritance of epigenetic modification, the subsistence of biochemical as well as epigenetic memories of stress, and their importance in the adaptation of plants to changing environmental conditions.

## 5. Conclusions

Although the evidence for the involvement of epigenetic modifications, particularly DNA methylation, in controlling the responses of plants to abiotic stresses as well as in stress memory is accumulating rapidly [48,50,76], only a few reports are present on biochemical stress memory in plants. Since abiotic stress causes several biochemical, physiological, and epigenetic changes in plants, our attempts to identify some of the biochemical (proline, total phenols, antioxidant potential, MDA) and epigenetic (5-mC) parameters associated with the mitigation of deleterious effects of abiotic stress over three generations could result in demonstrating their roles in stress memory. Our findings not only report on the role of biochemical and epigenetic factors in protecting the plant from stress, but also demonstrate the transgenerational inheritance of some of the stress-induced/acquired changes helpful for mitigating the deleterious effects of drought stress. To the best of our knowledge, this is the first report on the involvement of certain biochemical and epigenetic parameters in memorizing an episode of stress across the generations. Such epigenetic markers might be utilized in crop improvement programs through CRISPR/dCas9-based targeted DNA (de)methylation editing [77], which was used earlier to introduce a heritable late-flowering phenotype [78]. This opens up a new method for the genetic manipulation of plants utilizing the epi-allele/epimark in the development of climate-smart crop plants.

## Data Availability

All data are available in this manuscript.
